# The Influence of Heat Aging Treatments on the Cavitation Erosion Behavior of a Type 6082 Aluminum Alloy

**DOI:** 10.3390/ma16175875

**Published:** 2023-08-28

**Authors:** Ilare Bordeasu, Brândușa Ghiban, Lavinia Madalina Micu, Alexandru Nicolae Luca, Alin Mihai Demian, Dionisie Istrate

**Affiliations:** 1 Mechanical Machinery Equipment and Transport Department, University Politehnica of Timisoara, 300006 Timisoara, Romaniaalexandruluca03@gmail.com (A.N.L.); 2 Metallic Materials Science and Physical Metallurgy Department, University Politehnica Bucharest, 060042 Bucharest, Romania; 3 Department I—Agricultural Technologies, University of Life Sciences “King Mihai I” from Timișoara, 300645 Timisoara, Romania

**Keywords:** aluminum alloy 6082, cavitation erosion resistance, artificial heat treatment

## Abstract

It is known that a number of parts that operate in liquid media, such as the propellers of motorboats and pleasure river vessels, as well as the rotors of household pumps and the radiators and pumps in the cooling system of motor vehicles are made, as a rule, of aluminum-based alloy. Research during maintenance leads to the conclusion that, in certain operating conditions, due to the turbulent character of the flow, with pressure drops to below the vaporization level, it inevitably reaches the threshold of cavitation, which manifests itself through its effects, especially through erosion. To increase the lifetime, these alloys are currently subjected to techniques to improve the structure’s resistance to the cyclic stresses of cavitational microjets. Among these techniques are volumetric heat treatments, which lead to changes in the microstructure and mechanical property values, with an effect on the behavior and resistance to cavitation erosion. This paper studies the influence of heat aging treatments on the cavitation erosion behavior of an aluminum alloy type 6082, in the cast state. The heat treatments applied were 140 °C/1 h, 12 h, 24 h and 180 °C/1 h, 12 h, 24 h. The MDEmax and MDERs parameters were determined and a correlation could be made between the values of the mechanical-resilient characteristics and the resistance to cavitation erosion in the case of aluminum alloy 6082.

## 1. Introduction

The use of aluminum alloys in industrial fields is one of their widest applications, due to the low specific mass and the values of the mechanical properties that confer resistance to various mechanical and hydrodynamic stresses. In the automotive, agro-industrial, and naval fields, aluminum alloys are used for the manufacture of the most diverse equipment, from storage containers to irrigation systems, to cooling systems in the construction of hydro-pneumatic systems, to pumps of thermal engines of agricultural vehicles, to pump rotors from water wells, and to propulsion propellers of boats and pleasure vessels [1,2,3,4,5]. The lifetime of such parts, which work with liquids, is affected by the hydrodynamic flow regime, because through pressure variations, the flow becomes cavitational, when the local pressure drops below the value of the vaporization pressure [3,5,6,7,8,9,10]. Known as cavitation erosion in such situations, after a certain duration of operation, the surfaces of the parts in the cavitational field are subject to this process. Cavitation erosion is one of the manifestation effects of cavitation corrosion, well-known and developed in the specialized literature. This effect is mechanical in nature and consists in the creation of caverns due to ruptures produced in the structure of the surface material due to cyclic stresses (local fatigue) of impact pressures produced by microjets and shock waves developed by the implosion of cavitation bubbles. Considering the correlation structure—properties—performance, which is found in the field of materials science, cavitation erosion is a very important aspect among properties of engineering materials and their behavior in exploitation. As a result, extensive studies have begun to be carried out to increase the resistance of surface structures to the erosion produced by the cyclic stresses of cavitational microjets [10,11,12,13].

Aluminum alloys are widely used for properties such as mechanical behavior and corrosion resistance in aggressive media. Series 6xxx, the Al–Mg–Si system, is commonly used for high-stress applications, transport applications, bridges and cranes, the paper industry, and the petrochemical industry due to its high strength-to-weight ratio, good formability, and favorable corrosion resistance [14,15,16,17,18]. In particular, the corrosion resistance of Al–Mg–Si alloys has been proved to be better than that of Al–Zn–Mg and Al–Cu–Mg systems by various researchers [19,20,21,22,23] because of lower alloying degree and smaller potential difference between precipitates and matrix. Therefore, significant industrial interests have been attracted to the exact dependence between chemical compositions, thermo-mechanical history, and corrosion susceptibility of Al–Mg–Si alloys [24,25,26,27,28,29]. The precipitation sequence of 6xxx series alloys is as follows [30,31,32,33]: super saturated solid solution (SSSS) → solute clusters → GP zones (spherical) → β″ (needle) → β′ (rod) → β (Mg_2_Si). The metastable β″-phase is the primary strength phase under the peak aging process. To date, a number of studies on the aging behaviors of 6xxx series aluminum alloy have been carried out [23,24,25,26]. Michailidou et al. [34] investigated the effect of temperature range 140–225 °C on corrosion characteristics, which showed that with an increase in temperature, the corrosion type changed from IGC to pitting corrosion. Zaid et al. [35] proposed that the artificial aging introduced a susceptibility to IGC, and over-aging tended to increase the cathodic kinetics. It was documented that the GP zones gradually transform to β″ phase with the aging temperature from 180 °C to 330 °C [36]. Chen et al. [36] proposed that the dynamic behavior of grain boundary nanoparticles changes with aging temperature, and the evolution process of nanoparticles slows down or even does not evolve at low aging temperature. The most popular alloy type is 6082, which belongs to the Al-Mg-Si system alloys. Alloy 6082 has specific properties, including high tensile strength, high durability, anti-corrosive qualities, flexibility, good dimensional accuracy, fine surface finishes, and the ability to hold high temperature and heavy loads. The other features, like stress corrosion cracking resistance, pitting resistance, crevice corrosion resistance, and oxidation resistance, make it best for several industrial applications. It is typically formed by extrusion and rolling, but as a wrought alloy, it is not used in casting. It can also be forged and clad, but that is not common practice with this alloy. It cannot be work-hardened, but it is commonly heat-treated to produce tempers with a higher strength but lower ductility. Therefore, it is necessary to study the effect of higher aging temperature on microstructure and corrosion behavior of 6082 aluminum alloy. Consequently, this paper aims to study the influence of volumetric heat treatments, which bring about microstructural changes and mechanical property values, with an effect on increasing the resistance to the destructive impact forces of microjets and shock waves generated by the cavitation mechanism.

## 2. Materials and Methods

Materials used in the experimental research have the chemical composition indicated in Table 1, corresponding to an aluminum alloy from the 6082 series.

On the experimental samples in the T651 state, heat treatments of artificial aging were performed on experimental samples with dimensions 10 mm × 10 mm × 50 mm as follows: aging at 140 °C, with three maintenance periods, 1 h, 12 h, and 24 h; artificial aging at 180 °C, with three holding periods, 1 h, 12 h, and 24 h. The heat treatments were carried out in a Nabertherm furnace, within the Laboratory of Metallic Materials Science and the Physical Metallurgy Section of the Polytechnic University of Bucharest. For each type of heat treatment, six tests were performed to determine the mechanical properties: breaking strength, yield strength, elongation, toughness, hardness, and microhardness. Grain size determination was performed according to ASTM E3, ASTM E 407, ASTM E112, using Barker electrolytic reagent at ×100 power. Structural investigations were performed on an OLYMPUS microscope. The determination of the mechanical characteristics of the test samples subjected to different heat aging treatments was carried out on a universal testing machine Walter + Bai AD Switzerland model LFV 300. The impact resistance was carried out by determining the KCV values on the Pendulum Charpy hammer type Walter Bai with 300 J. Macroscopic structural analysis was performed on an OLYMPUS SZX stereomicroscope, equipped with QuickMicroPhoto 2.2 software, and microscopic analysis on a REICHERT UnivaR type optical microscope equipped with Image Pro Plus software. The analysis of the samples by X-ray diffraction was carried out with the D8 Discover diffractometer, Bruker-Germany, Cu anode tube (λ = 1.540598 Å), 1D Lynx Eye detector. The diffractograms were recorded with an angular increment of 0.040, at a scanning speed of 1 s/step, angular range of 2θ = 10–100°. Scanning electron microscopy analysis was performed on a Thermophischer Quattro S scanning electron microscope.

The experimental program, for cavitation testing of the structure’s resistance, took place in the Cavitation Erosion Research Laboratory of the Politehnica University of Timișoara, on the standard vibrating apparatus with piezoceramic crystals [6]. During the tests, the requirements described by the international standards ASTM G32-2016 [37], the laboratory custom [38,39], were followed, regarding the total duration of cavitation (165 min) and the 12 intermediate periods (each of 5 and 10 min and 10 of 15 min each), the one related to tracking the evolution of the destruction in the area of the cavity surface. The functional parameters of the vibrator, which determine the hydrodynamics of the vibratory cavitation, that is, the intensity of destruction, were rigorously controlled by a special software implemented in the computer connected to the vibrator. Their values, according to ASTM G32 standards, are:

- Double vibration amplitude = 50 µm;
- Vibration frequency = 20,000 ± 1% Hz;
- The power of the electric ultrasound generator = 500 W;
- Liquid medium = double distilled water;
- Liquid temperature = 22 ± 1 °C.

**Figure 1:**
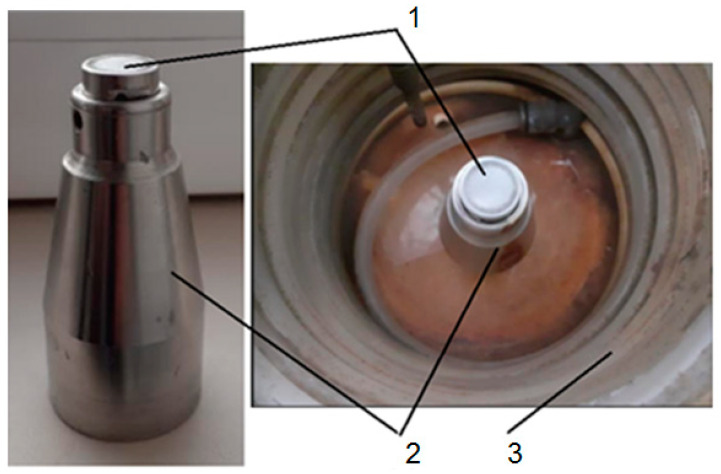
Sample fixture for testing by the indirect method. 1—sample for cavity testing (d = 15.8 mm, length = 18 mm); 2—sample fixing device for carrying out the experimental test; 3—the container with liquid and the cooling coil.

*Clarification:* The construction of the vibrating mechanical system is for the DIRECT TESTING METHOD (with the vibrating sample caught in the mechanical system), for metallic and non-metallic samples with a diameter of 15.8 mm and a length of 16 mm and with masses of 16–17.5 g. To generate erosion by vibrating cavitation, for light alloy samples, such as aluminum, due to the very low mass, even if the sample complies with the required geometric dimensions, the INDIRECT TESTING METHOD (stationary sample method) was used. In this sense, a special sample fixation system was built, as can be seen in Figure 1.

According to laboratory custom, in order to comply with the requirements of ASTM G32-2016, in order to reduce the influence of the surface quality on the results, before starting the test, the surface that was to be exposed to cavitation was polished to a roughness Ra = 0.2…0.8 µm [6]. From each type of state (semi-finished and with heat treatment), as a certainty of the accuracy of the experiment, as required by customs, three samples were tested. Before the start of the test and after each intermediate period of time, the eroded surface of each sample was photographed with the Canon Power Shot A480 and analyzed with the OPTIKA Vision Pro3 microscope from the Cavitation Erosion Research Laboratory, with the aim of highlighting and understanding the way degradation by erosion, that is, the mechanical response process of the surface structure to the cyclic stresses of microjets and shock waves generated by the implosion of cavitation bubbles.

## 3. Experimental Results, Interpretation, and Discussions

### 3.1. The Mechanical Behavior of the Experimental Samples

**Figure 2:**
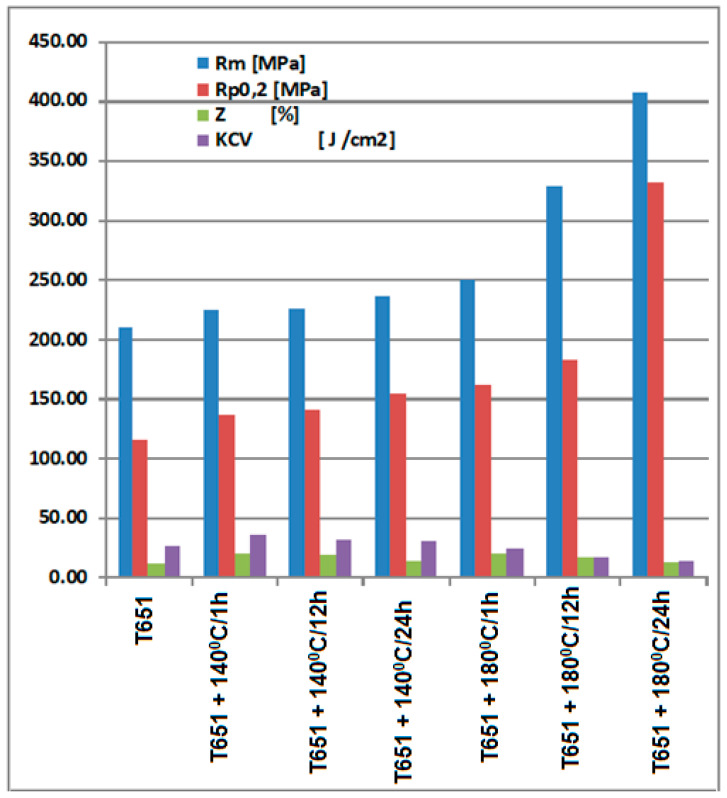
The evolution of the mechanical characteristics of the experimental specimens made of aluminum alloy type 6082, in different structural states.

For ease of presentation, description, analysis, and understanding of the research results, the symbols used to mark the samples, depending on the nature of the semi-finished alloy and the parameters of each heat treatment regime (temperature, holding time, cooling mode), are as follows:

U—state of the laminated semi-finished product (delivery state), without other heat treatments;

UP—state obtained by artificial aging at 160 °C, with a holding time of one hour and cooling in the oven;

UL—state obtained by artificial aging at 160 °C, with 12 h holding time and oven cooling;

UI—state obtained by artificial aging at 160 °C, with 24 h holding time and cooling in the oven;

UOP—state obtained by artificial aging at 140 °C, with a holding time of one hour and cooling in the oven;

UOL—state obtained by artificial aging at 140 °C, with 12 h holding time and cooling in the oven;

UOI—state obtained by artificial aging at 140 °C, with 24 h holding time and cooling in the oven;

The results regarding the mechanical behavior of the experimental samples are shown in Table 2, and suggestively represented in Figure 2.

A careful analysis of the experimental data allows the formulation of the following observations.

By applying heat aging treatments to aluminum alloy type 6082 products, in T651 condition, either at 140 °C or at 180 °C, with different holding times, there is an increase in mechanical strength, yield strength, and a decrease in cooking and resilience, a fact that allows a prediction of the increase in resistance to cavitational erosion. Thus, when applying an aging at 140 °C, the breaking strength can increase by 7–13% (differentiated according to the duration of holding at the temperature), the yield strength increases by 18–34%, the necking increases by 17–77%, and the resilience decreases by about 18–36%; when aging at 180 °C, the breaking resistance can increase by 18–94% (differentiated according to the duration of holding at the temperature), the yield strength increases by 39–186%, and the resilience decreases by about 4–43%. The explanation of the increase in mechanical characteristics is given by the precipitation of intermetallic compounds, in the metal matrix of solid solution α, precipitation that becomes abundant when the duration of maintenance at the aging temperature of 180 °C increases. This is highlighted by the structural analysis, both with the optical microscope and the X-ray diffraction analysis, presented in subchapter 3b.

**Figure 3:**
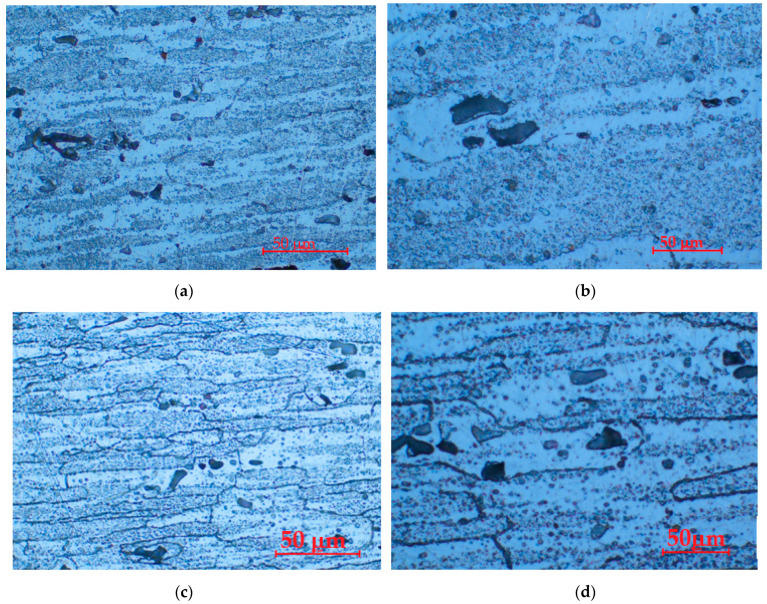

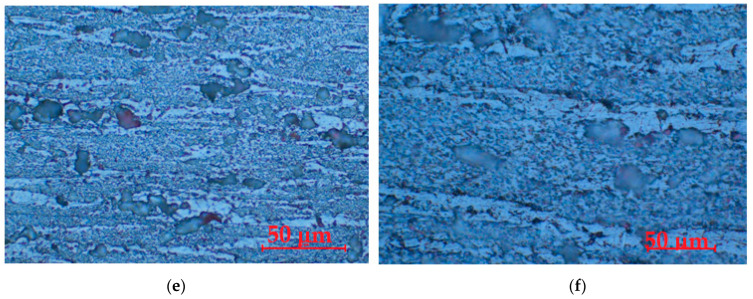
Microstructural aspects of the type 6082 aluminum alloy samples, in different structural states: (**a**,**b**)—state T651 (control sample); (**c**,**d**) state T651 + aging at 140 °C/24 h (UOI sample); (**e**,**f**)—T651 state + aging at 180 °C/24 h (UI sample); (**b**,**d**,**f**) detail images of (**a**,**c**,**e**).

In Figure 3b,d, we show the structure after applying aging at 140 °C for the longest holding time, 24 h, and in Figure 3e,f, the structure is highlighted after applying aging at 180 °C at the longest holding time, 24 h. It is thus noted that at a temperature of 180 °C and a duration of 24 h, there is the most abundant precipitation of intermetallic compounds in the solid solution matrix α, a fact that explains the almost doubling of the mechanical strength and yield strength, compared to the control sample, in T651 condition, not subject to aging.

**Figure 4:**
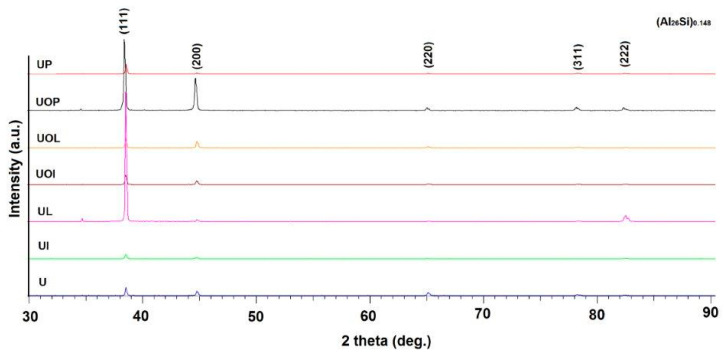
X-ray diffraction images of type 6082 aluminum alloy specimens in different structural states.

The X-ray diffraction analysis in Figure 4 confirms the results of the metallographic analysis, in the sense that by applying the aging treatments, the lattice parameter increases considerably, a sign of the hardening of the solid solution α.

### 3.2. The Cavitational Erosion Behavior of the Experimental Specimens

**Figure 5:**
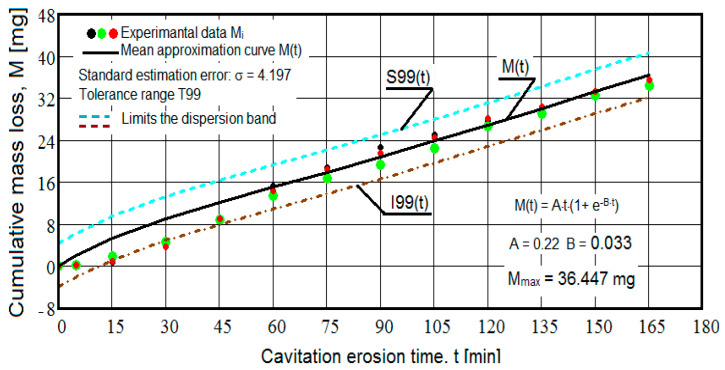
Variation of the cumulative eroded mass (control sample U).

**Figure 6:**
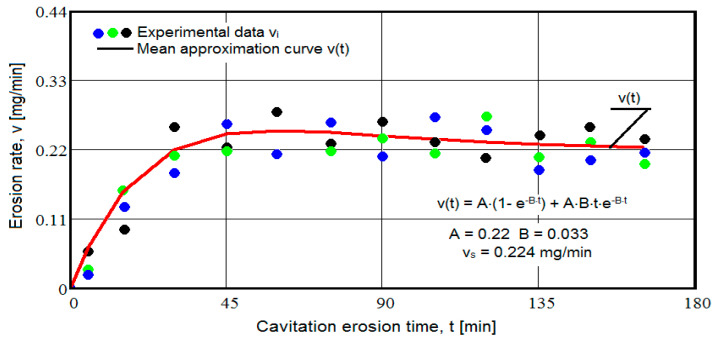
Variation of erosion speed (control sample U).

**Figure 7:**
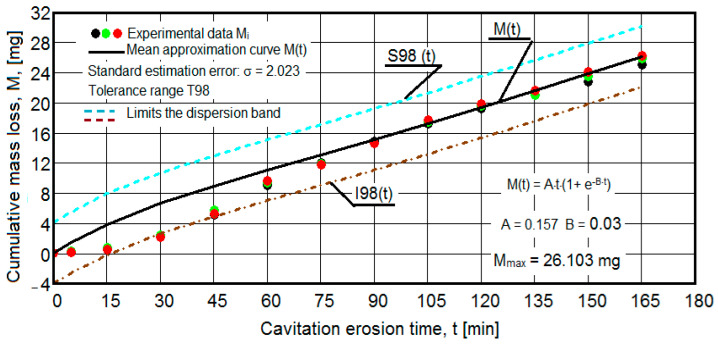
Variation of the cumulative eroded mass (sample UP).

**Figure 8:**
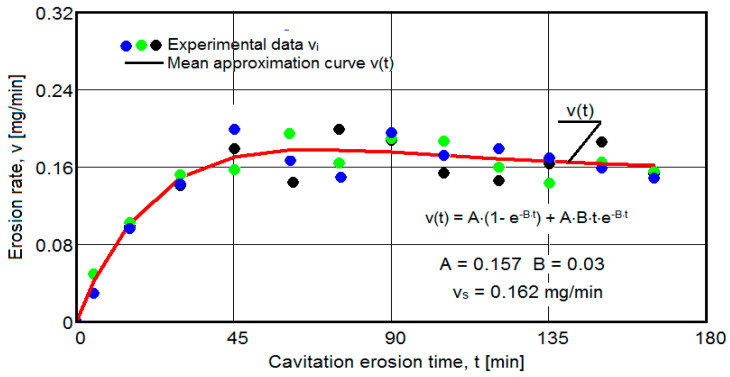
Variation of erosion speed (sample UP).

**Figure 9:**
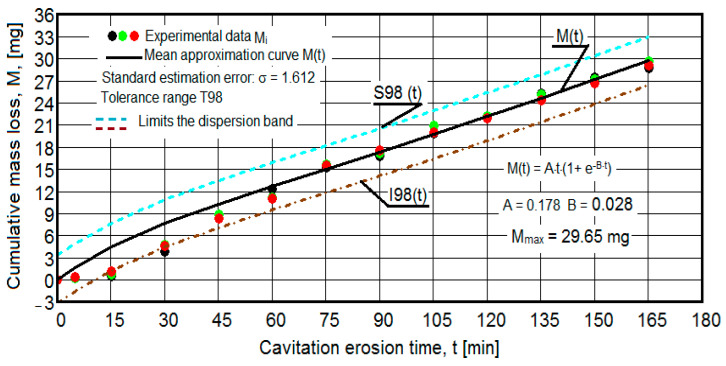
Variation of the cumulative eroded mass (sample UL).

**Figure 10:**
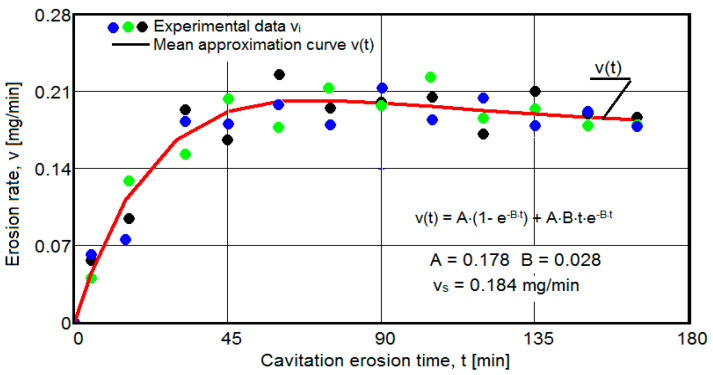
Variation of erosion speed (sample UL).

**Figure 11:**
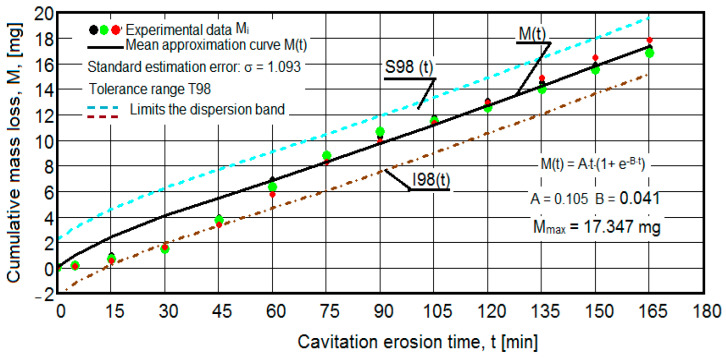
Variation of the cumulative eroded mass (sample UI).

**Figure 12:**
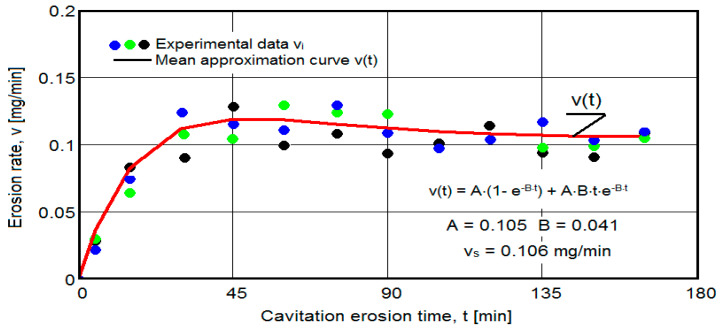
Variation of erosion speed (sample UI).

**Figure 13:**
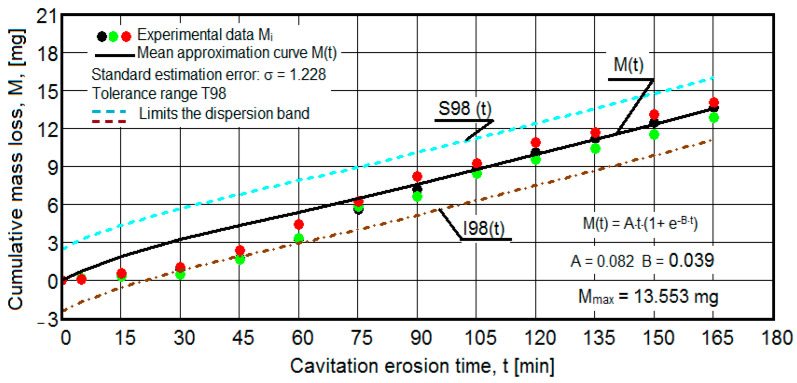
Variation of the cumulative eroded mass (sample UOP).

**Figure 14:**
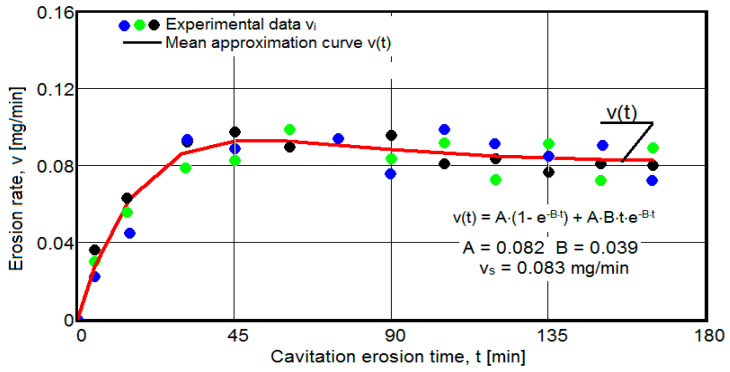
Variation of erosion speed (sample UOP).

**Figure 15:**
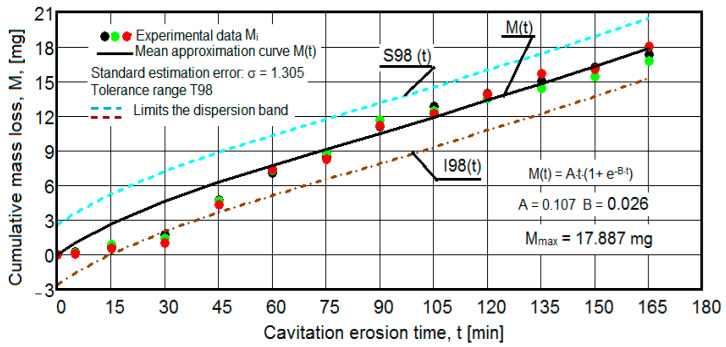
Variation of the cumulative eroded mass (sample UOL).

**Figure 16:**
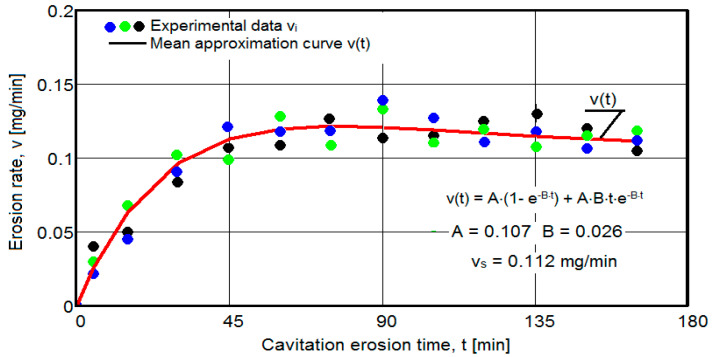
Variation of erosion speed (sample UOL).

**Figure 17:**
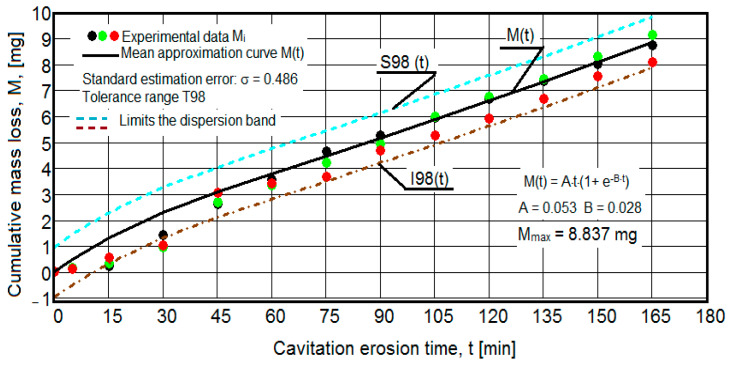
Variation of the cumulative eroded mass (sample UOI).

**Figure 18:**
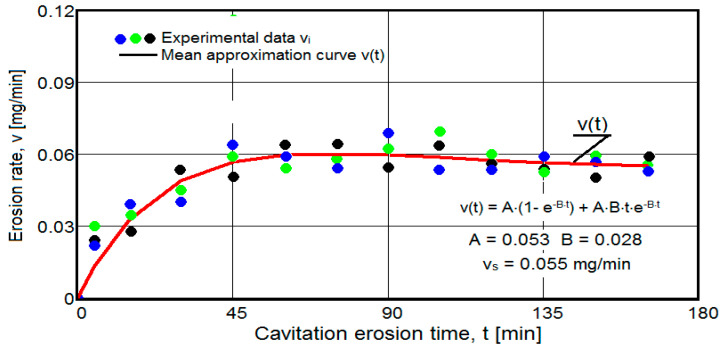
Variation of erosion speed (sample UOI).

The results of the cavitation test are expressed by the diagrams in Figure 5, Figure 6, Figure 7, Figure 8, Figure 9, Figure 10, Figure 11, Figure 12, Figure 13, Figure 14, Figure 15, Figure 16, Figure 17 and Figure 18, which contain the experimental values of the three samples (points in color) tested from each state of heat treatment and the specific averaging/approximation curves, which give the variation of the cumulative mass lost by erosion M(t) and of the average mass loss velocity v(t) in the intermediate period lasting 5, 10, and 15 min. These curves are the basis of the characterization of the behavior and resistance of the surface structure to the erosive stresses of the vibrating cavitation microjets and are built with the analytical relationships established by Bordeasu and collaborators [38] within the Cavitation Erosion Research Laboratory. Their forms, expressed by the analytical relations (1) and (2), are determined from the condition that the approximation ensures a tolerance interval ≤10%, of the dispersion of the experimental values of the cumulative mass against the M(t) curve [6].

- For the cumulative eroded mass:

M(t) = A·t·(1 − e^−B·t^) sau M(t) = A·t·(1 + e^−B·t^). (1)

- For the average erosion rate:

v(t) = A·(1 − e^−B·t^) + A·B·t·e^−B·t^ (2)

where

A is the scale parameter, established from the condition that the deviations of the experimental points from the curve are minimal;
B is the shape parameter of the curve.

In order to certify the accuracy of the test on the three samples, in the diagrams showing the variation of the mass loss M(t) with the duration of exposure to cavitation, the upper and lower tolerance intervals, that is, the area of the dispersion band of the experimental points, calculated with the Mathcad program, were delimited. The statistical relationships used have the following forms [39]:

- the equation of the exponential regression curve (rel.1);
- standard error of estimation:

(3) $$\sigma_{MDE}={\left[\frac{\sum_{i=0}^{12}{\left(M_{i}-M{\left(t\right)}_{i}\right)}^{2}}{n-1}\right]}^{\frac{1}{2}}$$

where

- M(t)_i_ is the mass of material lost through erosion defined by the averaging curve (rel. 1) at time t_i_;
- M_i_ is the average experimental value of the values obtained by experiment at time ti, on the three samples.

As a result of the complexity of the hydrodynamics of cavitation and the dependence of the resistance of the required surface structure on a multitude of factors (microstructural defects, type of microstructure, chemical composition, physical–mechanical properties), the tolerance ranges accepted for this type of stress (≤10%) range from 99% to 90% [39], whose upper “S” and lower “I” limits are calculated as follows:

- for the 99% tolerance interval:

S99(t) = M(t) + σ; I99(t) = M(t) − σ(4)

- for the 90% tolerance interval:

S90(t) = M(t) +10·σ; I90(t) = M(t) − 0·σ(5)

where σ is the average standard deviation of the experimental values from the averaging curve M(t).

Clarification: The diagrams in Figure 5, Figure 6, Figure 7, Figure 8, Figure 9, Figure 10, Figure 11, Figure 12, Figure 13, Figure 14, Figure 15, Figure 16, Figure 17 and Figure 18, through the evolutions of the averaging curves, the dispersion of the experimental values, in different intervals, compared to the averaging curves, show the behavior and resistance of the surface structure to the cyclic stresses of microjets and shock waves generated by vibrating cavitation. The values of the parameters Mmax (the mass lost through erosion, defined by the curve M(t) at 165 min) and vs (the value of the speed from 165 min defined by the curve v(t), known in the specialized literature as the stabilization speed of erosion, or the value towards which the v(t) curve asymptotically decreases [6,9,38,39,40]) are indicators of the structure’s resistance to cavitation stress, and by comparison, they serve to identify the material (structure) with the best resistance and behavior to cavitation erosion generated in the device used [6,9,39].

It should be noted that, according to all the studies in this field, mentioned in the specialized literature [39], the dispersions of the experimental values, the evolution forms of the averaging curves, and the values of the indicated parameters are an effect of the nature of the semi-finished product, of the parameters of the heat treatment regimes (treatment type, temperatures, durations), and of the microstructure and mechanical properties (hardness, mechanical resistance to breaking, resilience, etc.).

**Figure 19:**
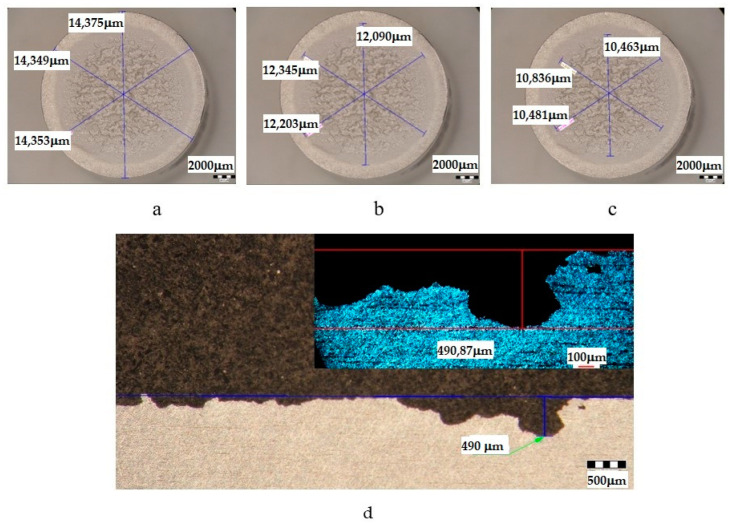
Analysis of the surfaces subjected to cavitational erosion of the control sample (T651 condition) made of aluminum alloy type 6082: (**a**)—measurement of the outer diameter; (**b**)—measuring the diameter of the surface affected by erosion; (**c**)—measuring the diameter of the surface most affected by erosion; (**d**)—measuring the maximum penetration depth of cavitational erosion (background image with depth measured by stereomicroscope, top right image with depth measured by optical microscope).

**Figure 20:**
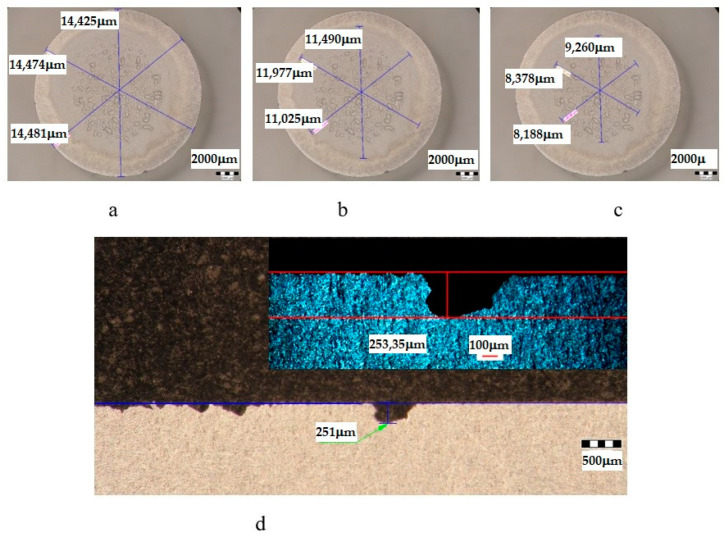
Analysis of the surfaces subjected to cavitational erosion of the T651 + condition samples aging at 140 **°**C/1 h (UOP), from aluminum alloy type 6082: (**a**)—measuring the outer diameter; (**b**)—measuring the diameter of the surface affected by erosion; (**c**)—measuring the diameter of the surface most affected by erosion; (**d**)—measurement of the maximum penetration depth of cavitational erosion (background image depth measured with a stereomicroscope, top right image with depth measured with an optical microscope).

**Figure 21:**
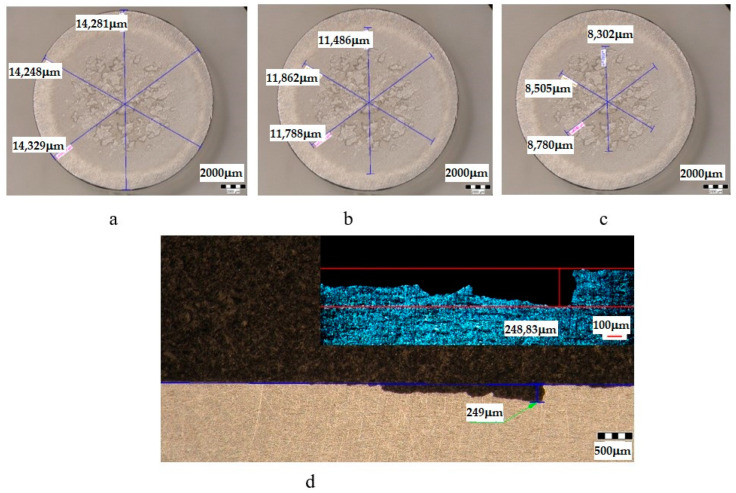
Analysis of the surfaces subjected to cavitational erosion of the T651 + condition samples aging at 140 **°**C/12 h (UOL), from aluminum alloy type 6082: (**a**)—measuring the outer diameter; (**b**)—measuring the diameter of the surface affected by erosion; (**c**)—measuring the diameter of the surface most affected by erosion; (**d**)—measurement of the maximum penetration depth of cavitational erosion (background image depth measured with a stereomicroscope, top right image with depth measured with an optical microscope).

**Figure 22:**
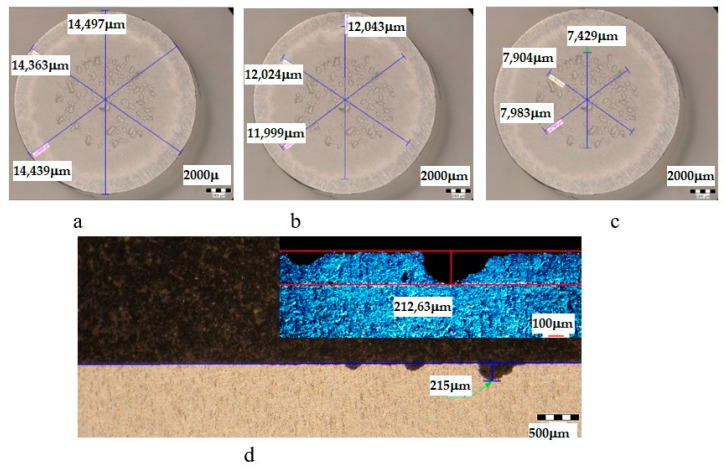
Analysis of the surfaces subjected to cavitational erosion of the T651 + condition samples aging at 140 **°**C/24 h (UOI), from aluminum alloy type 6082: (**a**)—measuring the outer diameter; (**b**)—measuring the diameter of the surface affected by erosion; (**c**)—measuring the diameter of the surface most affected by erosion; (**d**)—measurement of the maximum penetration depth of cavitational erosion (background image depth measured with a stereomicroscope, top right image with depth measured with an optical microscope).

**Figure 23:**
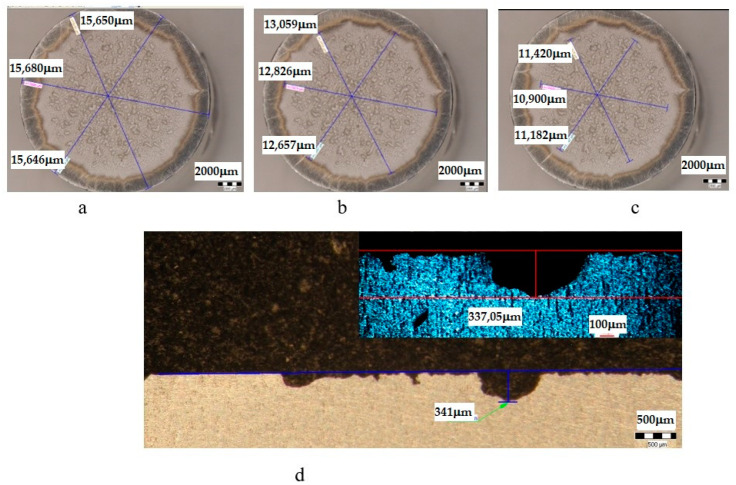
Analysis of the surfaces subjected to cavitational erosion of the T651 + condition samples aging at 180 **°**C/1 h (UP), from aluminum alloy type 6082: (**a**)—measuring the outer diameter; (**b**)—measuring the diameter of the surface affected by erosion; (**c**)—measuring the diameter of the surface most affected by erosion; (**d**)—measurement of the maximum penetration depth of cavitational erosion (background image depth measured with a stereomicroscope, top right image with depth measured with an optical microscope).

**Figure 24:**
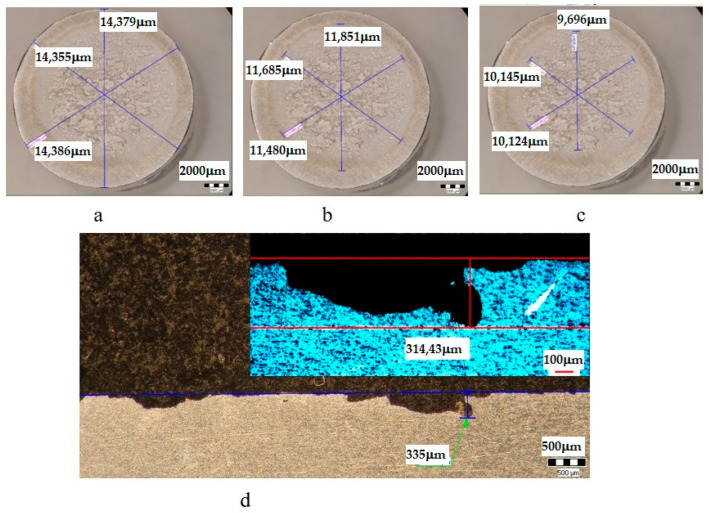
Analysis of the surfaces subjected to cavitational erosion of the T651 + condition samples aging at 180 **°**C/12 h (UL), from aluminum alloy type 6082: (**a**)—measuring the outer diameter; (**b**)—measuring the diameter of the surface affected by erosion; (**c**)—measuring the diameter of the surface most affected by erosion; (**d**)—measurement of the maximum penetration depth of cavitational erosion (background image depth measured with a stereomicroscope, top right image with depth measured with an optical microscope).

**Figure 25:**
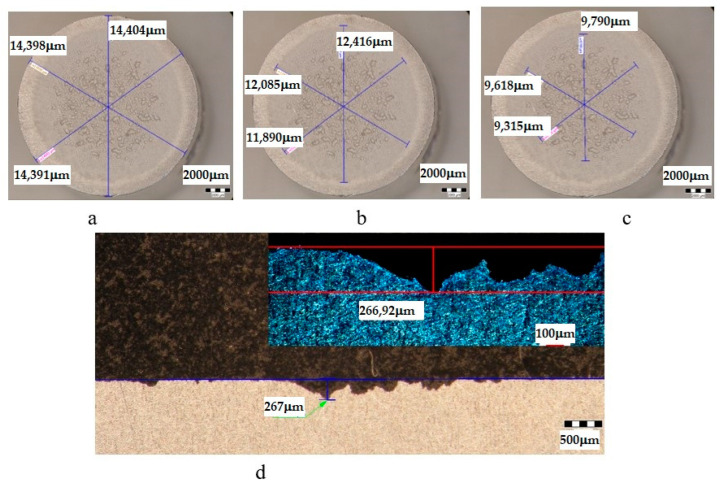
Analysis of the surfaces subjected to cavitational erosion of the T651 + condition samples aging at 180 **°**C/24 h (UI), from aluminum alloy type 6082: (**a**)—measuring the outer diameter; (**b**)—measuring the diameter of the surface affected by erosion; (**c**)—measuring the diameter of the surface most affected by erosion; (**d**)—measurement of the maximum penetration depth of cavitational erosion (background image depth measured with a stereomicroscope, top right image with depth measured with an optical microscope).

The metallographic stereomicroscope analysis of the cavitationally eroded surfaces allowed the fractographic evaluation, as well as the definition of the production mechanism of this phenomenon. The results of the stereomicrostructural analysis are shown qualitatively in Figure 19, Figure 20, Figure 21, Figure 22, Figure 23, Figure 24 and Figure 25 and quantitatively in Table 3. In the control sample, Figure 18, an attack generated by aggressive cavitational erosion is noted, with macroscopically visible caverns, located on a large surface, about 85% of the entire surface exposed to erosion, and the most stressed surface has an extension of 74% (as can be seen from Table 3, which centralizes the quantitative results presented in Figure 18). The maximum depth of corrosion penetration reaches 490 μm (Figure 19d), a depth that is considered very high, placing this alloy in the framework of metallic materials with low resistance to cavitational erosion. When applying the aging treatment at 140 °C, regardless of the holding time, a slight improvement in the resistance to cavitational erosion is observed (Figure 20, Figure 21 and Figure 22), in the sense of the decrease in the surface most affected by the corrosive attack, from 71% (at a 1 h hold) to 69% (at a 12 h hold), reaching 66% (at a 24 h hold). Also, the maximum penetration depth of the corrosive attack also registers a decrease compared to the control sample from 337 μm (after a 1 h hold, Figure 20d) to 314 μm (after a 12 h hold, Figure 21d), reaching 267 μm (after a 24 h hold, Figure 22d). It is noteworthy that, although the values obtained after the application of the aging heat treatment, both of the attacked surfaces and of the penetration depth, are high, they maintain the alloy in the assessment of a low resistance to cavitational erosion. When applying the aging treatment at 180 °C, regardless of the holding time, a considerable improvement in the resistance to cavitational erosion is noted (Figure 23, Figure 24 and Figure 25), both compared to the control sample and compared to the samples aged at 140 °C. Thus, the surface area most affected by the corrosive attack decreases, to 60% (after 1 h and 12 h maintenance) and to 54% (after 24 h maintenance). Also, the maximum penetration depth of the corrosive attack also registers a decrease compared to the control sample, to 253 μm (after a 1 h hold, Figure 19d) and to 249 μm (after a 12 h hold, Figure 21d).

### 3.3. Scanning Electron Microscope Analysis of Cavitationally Eroded Samples

**Figure 26:**
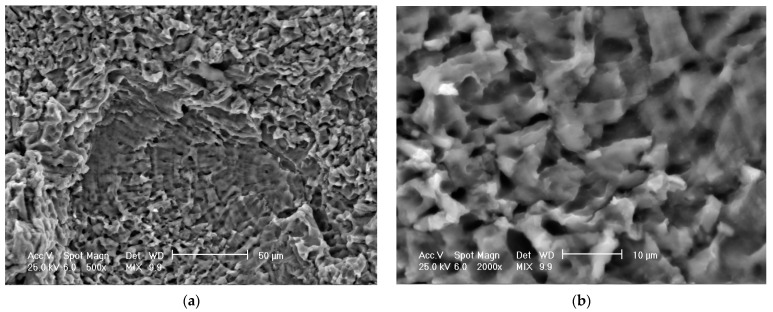

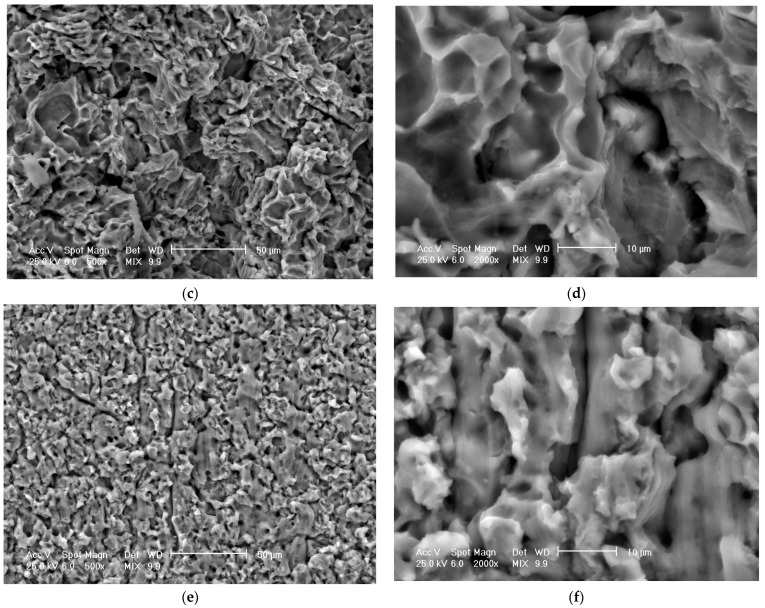
SEM images of the surfaces subjected to cavitational erosion of type 6082 aluminum alloy specimens, in different structural states: (**a**,**b**)—control sample; (**c**,**d**)—UOI sample (T651 state + aging at 140 °C/24 h); (**e**,**f**)—UI sample (T651 condition + aging at 180 °C/24 h), (**b**,**d**,**f**—details of images **a**,**c**,**e**).

The results of the scanning electron microscope analysis are shown in Figure 26. It is noted that in all investigated samples, the corrosive attack is very intense, both in the control sample (Figure 26a,b) and in the samples subjected to the aging treatment, either at 140 °C 24 h (Figure 26c,d) or 180 °C/24 h (Figure 26e,f). The attack is intergranular, with a brittle appearance, faceted cleavage, and propagation of the intergranular fracture front. The similarity of the aspects, even if different values of the cavitational erosion penetration depth are recorded, show the same manifestation of the intergranular erosion phenomenon, specific to metallic materials with low resistance to this form of corrosion.

## 4. Discussion—Discussions and Interpretations of the Experimental Results Regarding the Behavior and Resistance to Cavitation

The diagrams characteristic of the erosion generated by the shock waves and the microjets resulting from the implosion of the cavitational bubbles formed in the process of vibrating cavitation are shown in Figure 5, Figure 6, Figure 7, Figure 9, Figure 11, Figure 13, Figure 15 and Figure 17, which contain data that allowed the analysis of the behavior and strength of the structure of the types of material states after the variation of cumulative mass losses with the duration of exposure to cavitation. Figure 6, Figure 8, Figure 10, Figure 12, Figure 14, Figure 16 and Figure 18 contain data that allowed the analysis of the behavior and resistance of the structure of the types of material states after the variation of the erosion speed (the average speed of losses of mass during the intermediate duration of the cavitation attack) with the duration of exposure to cavitation.

The data from the diagrams presented in Figure 5, Figure 6, Figure 7, Figure 9, Figure 11, Figure 13, Figure 15 and Figure 17 lead us to the following findings:

- The values of the tolerance fields (between 97% and 99%), related to the dispersion bands, justify the accuracy of the test program, through the rigorous control of the parameters of the vibrator that dictate the intensity of the destruction by cavitation, regardless of the state (semi-finished or with heat treatment); the structures of the three samples, during exposure to cavitation, have a similar behavior, the values of the standard error σ tending to decrease from 4.197 (for the U-state of the semi-finished product, for which the average algebraic cumulative mass loss is 36.447 mg) to 0.486 (for the UOI condition, for which the cumulative algebraic average mass loss is 8.837 mg), with increased resistance to cyclic cavitation stresses.
- A time interval exists between 0 and 30 min, depending on the state of the sample, in which the mass losses are reduced, following that from here until the end (165 min), the cumulative mass loss is, as is natural, continuously increasing. The small losses in the first part show the dependence of the surface resistance, at the impact pressures with the microjets of the structure, on the grain sizes and on the number and sizes of intermetallic compounds.

Obviously, this resistance, according to older studies by Garcia a.o [40], in the initial phase of the generation of networks of cracks and breaks with expulsions, is primarily dependent on the hardness, but also on the values of the mechanical properties (see Table 2), which determines the level of intercrystalline bonding forces. However, unlike ferrous alloys, with very high resistance to cavitation erosion, such as stainless steels with martensitic structures and surfaces hardened by various technologies [8,9], where hardness is the determining factor in surface resistance to the demands of cavitational microjets, in the case of these samples, as shown by the values of the MDEmax parameter (the highest for the UOI sample (8837 mg—Figure 16) and the lowest for the U sample (36,447 mg—Figure 5)), this leads to the opinion that the strength of the alloy structure is strongly dependent on the mechanical properties that determine high capacity for plastic deformation (resilience).

- The decrease in the slopes of the M(t) curves, after 30 (60) min, shows the beneficial effect of the heat treatment regime on the resistance of the structure to the stresses of cavitational microjets, the highest slope being for the state of delivery (sample U), and the lowest for the UOI sample (heat treatment carried out at 140 °C lasting 24 h).
- At certain stress durations, the differences between the experimental values of the three tested samples are small, at certain times even insignificant (for sample U 45: 120 min, 135 min, 165 min; for sample UP: 30 min, 75 min, 90 min, 105 min, 135 min; for the UL test: 45 min, 75 min, 120 min, 165 min; for the UI test: 30 min, 45 min, 75 min; for the UOP test: 15 min, 75 min, 105 min; for the UOL sample: 15 min, 60 min, 120 min; for the UOI sample: 15 min, 60 min, 105 min, 135 min), which shows the effect of the heat treatment, from the point of view of structural homogeneity and dispersion of mechanical property values in the structure of the required surfaces, but also in the volume of samples.

The data from the diagrams shown in Figure 14, Figure 16, Figure 18, Figure 20, Figure 22, Figure 24 and Figure 26 lead us to the following findings:

- Similar conclusions can be reached as the analysis carried out on the diagrams that showed the variation of mass losses with the duration of cavitation, that is, small, natural differences between the experimental values of the three samples, from each state.
- The dispersion of the experimental values, at various attack times, shows that there are some differences between the surface structures of the three samples, caused, in particular, by the structural defects, by the intermetallic compounds and the sizes of the ejected grains. Overall, the tendency of the behavior of the structures of the three samples to the vibratory cavitation stresses is close, a fact confirmed by the evolution of the v(t) curve.
- After 45 (60) min, the averaging/approximation curves v(t) decrease asymptotically towards the value vs. This evolution is determined by the decrease in mass losses (see the related diagrams) as a result of the reduction in the energy absorbed to break the bonds between the grains and the expulsion.

From our studies [2,7,9], this decrease is caused by the mechanical hardening over time of the superficial layer, by the number and geometric dimensions of the caverns in which, during the compression phase of the sonotrode, air enters that acts as a shock absorber in the expansion phase of the sonotrode, a phenomenon that mitigates the impact force of the cavitational microjets.

- The evolution of the curve v(t), from the maximum value to the minimum value, is slower for structures with high resistance (see UOI sample, Figure 18).

**Figure 27:**
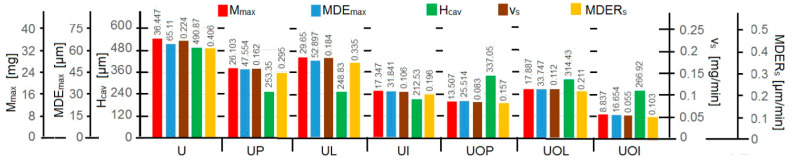
Histogram of the evaluation of the resistance of the structure to cavitation erosion.

To evaluate the resistance, the histogram in Figure 27 compares the values of the parameters recommended by ASTM G32 standards and used in the Cavitation Erosion Research Laboratory custom.

The parameters MDEmax and MDERs were calculated with the following relation:

(6) $${\mathrm{MDE}}_{\max}=\frac{4\cdot M_{\max}}{\rho \cdot \pi \cdot d_{p}^{2}}\;\mathrm{respectiv}\;{\mathrm{MDER}}_{s}=\frac{4\cdot v_{s}}{\rho \cdot \pi \cdot d_{p}^{2}}$$

where

d_p_ = 15.8 mm represents the diameter of the surface exposed to cavitation.

The diagram also shows the parameter H_cav_, which represents the value of the maximum depth of the cavern, measured with a stereomicroscope (see Figure 19d, Figure 20d, Figure 21d, Figure 22d, Figure 23d, Figure 24d and Figure 25d). Mentioning this depth is intended to prove that its value is dependent on the arbitrary place where the section for measurement was made and that, compared to MDE_max_, which is an average value on the cavitated surface, it cannot, in any case, serve as a parameter credible in comparing the resistance of a structure to cavitation erosion.

The histogram in Figure 27 shows that, regardless of the parameter we want to refer to (including H_cavb_), all heat treatment regimes led to higher strengths than that obtained for sample U (corresponding to the state of delivery (laminated semi-finished product)). The resistance increases compared to the U condition, regardless of the parameter used, being from 20% (UL sample) to more than 4 times (UOI sample). This leads us to support the necessity of volume heat treatments in increasing the cavitation resistance of the structure of aluminum-based alloys such as alloy 6082.

After the heat treatment regime, we find that the structure of the UOI sample (temperature of 140 °C and holding time of 24 h) confers the highest resistance to cavitation erosion. Among all the treatment regimens, the lowest resistance was recorded for the UL sample (temperature of 180 °C and duration of 12 h)

Although they are regimes with different temperatures and durations, the treatments applied to the UI and UOL samples show that the differences between the values of the mentioned parameters, regardless of which we report (M_max_, MDE_max_, v_s_ or MDER_s_), are about 1%. This aspect confirms that different volume heat treatment regimes can create structures with similar cavitation erosion resistances.

Among the regimes with the temperature of 180 °C, the values of the parameters in the histogram indicate the one with a duration of 24 h (sample UI) as the one that leads to the structure and mechanical properties that determine the best resistance to cavitation erosion (higher than the strength of the sample structure UP by about 53% according to the values of M_max_, MDE_max_, v_c_, and MDER and by about 19% according to the values of H_cav_, and compared to the strength of the sample structure, UL is higher by about 70% after M_max_ and MDE_max_, by about 73% after vs and MDER_s_, and by about 17% after H_cav_).

Among the regimes with a temperature of 140 °C, the values of the parameters in the histogram also indicate the one with a duration of 24 h (the UOI sample) as being the one that leads to the structure and mechanical properties that determine the best resistance to cavitation erosion (higher compared to the strength of the structure of the sample UOP by 53% according to the values of M_max_ and MDE_max_, by about 51% according to the values of vs and MDER_s_, and by 26% according to the values of H_cav_, and compared to the strength of the structure of the sample, UOL is about 2 times higher according to the values of M_max_, MDE_max_, v_c_, and MDER_s_ and by about 17% according to the values of H_cav_).

The fact that for both temperatures the holding time of 24 h led to structures with high strengths suggests that of the three times (1 h, 2 h, and 24 h), this is the one that can be used for parts made of alloy 6082, type pump rotors, cooling radiators, and boat propellers, which will be operated in cavitation mode.

## 5. Conclusions

The experimental research carried out in the present work allowed the formulation of the following conclusions:

- The structure of the alloy in the T651 state is constituted by solid solution α in which fine compounds of Mg_5_Si_6_ dispersed in the matrix are evident. By applying heat aging treatments, the structural aspect does not change much, the amount of compounds increasing considerably.
- Applying heat aging treatments to aluminum alloy type 6082 products can lead to an increase in mechanical strength, yield strength, and a decrease in necking and resilience, as follows. When aging at 140 °C, the breaking strength can increase by 7–13% (differentiated according to the duration of holding at the temperature), the yield strength increases by 18–34%, the necking increases by 17–77% and the resilience decreases by about 18–36%; when applying an aging at 180 °C, the fracture resistance can increase by 18–94% (differentiated according to the duration of holding at the temperature), the yield strength increases by 39–186%, and the resilience decreases by about 4–43%.
- The dispersion mode of the experimental values compared to the averaging curves M(t) and v(t), as well as the evolutions of the two curves, show similar and distinct/specific elements of the behavior of the surface structure to the erosion created by the cyclic stresses of the microjets produced by vibratory cavitation. Basically, these diagrams show the effect of the volumetric heat treatment regime (temperature and holding time) on the mechanical response to cyclic fatigue stresses, through the structures and values of the mechanical properties obtained.

The conclusions drawn from the experimental research and the analysis of the results obtained, based on the specific parameters and the photographic images from significant times, as well as the microscopic ones from the end of the cavitation test, are as follows:

- The cavitation resistance of the structures resulting from artificial aging heat treatments, regardless of the parameters of the heat treatment regime, is superior to the structure of the control sample, cast (sample U) from 30% to more than 4 times according to the values of the M_max_ and MDE_ma_ parameters and from 21% to more than 4 times according to the values of the parameters v_s_ and MDER_s_.
- For all samples, regardless of whether it is blank or thermally treated, in the first 15 (30) min of vibratory cavitation, material losses are reduced due to compliance with the specific erosive mechanism, through which, in the attacked surface, more elasto-plastic deformations and networks are produced of cracks and the tips of roughnesses and abrasive dust are removed. Material ejections, with the creation of pittings, are significantly reduced in the mass values recorded by weighing.
- In all samples, starting from minute 30 (45) and until the end of the test, the losses are of close order, which is why the curve M(t) becomes linear, the differences between the experimental values of the speeds are small, and the curve v(t) decreases asymptotically towards the stabilization value v_s_.
- In all samples, the evolutions of the v(t) averaging curves have an asymptotic tendency towards stabilization at the maximum value (v_s_). Based on previous experiences, we believe that this mode of evolution is the effect of the mechanical properties, of the hardening over time, of the applied layer and of the reduction in the stress/impact force, as a result of the cushioning effect of the air penetrating into the formed caverns.
- The thermal treatment regime by artificial aging that gives the best resistance to vibrating cavitation erosion is the one with a temperature of 140 °C and a holding time of 24 h.
- The thermal treatment regime that led to the structure with the lowest resistance to cavitation is the one with a temperature of 180 °C and a holding time of 12 h.
- The differences between the experimental values and their dispersions in front of the averaging curves are expressions of the influence of the structure on the resistance to cavitation by the regime parameters of the heat treatment, by the microstructural changes, and by the values of the mechanical properties, a fact confirmed by the microscopic and photographic images from the significant times.
- The increase in resistance to cavitation, conferred by heat treatment regimes, justifies the possible use of aluminum alloy type 6082 in order to expand applications to parts that work in cavitation regimes, such as pump rotors and boat propellers.

## Figures and Tables

**Table 1:** Chemical composition of the experimental aluminum alloy.

Alloy	Chemical Composition, %wt.								
	Si	Fe	Cu	Mn	Mg	Cr	Zn	Ti	Al
**Experimental**	1.25	0.31	0.09	0.62	0.86	0.17	0.088	-	rest
**EN AW-AlSi1MgMn**	0.7–1.3	Max. 0.5	Max. 0.1	0.40–1.0	0.6–1.2	Max. 0.25	Max. 0.2	Max. 0.1	rest

**Table 2:** The mechanical characteristics of the experimental samples made of aluminum alloy type 6082.

Sample	State	Mechanical Characteristics			
		Fracture Strength, MPa	Yield Strength, MPa	Necking, %	Resilience, KCV, KJ
**U-**	control-T651	210	116	11.6	25.0
**UOP**	T651 + 140 °C/1 h	225	137	20.5	34.7
**UOL**	T651 + 140 °C/12 h	236	141	18.5	30.7
**UOI**	T651 + 140 °C/24 h	236	155	13.6	29.7
**UP**	T651 + 180 °C/1 h	250	162	20.0	24.0
**UL**	T651 + 180 °C/12 h	329	182	16.0	16.3
**UI**	T651 + 180 °C/24 h	407	332	12.0	14.2

**Table 3:** Quantitative parameters of the experimental samples subjected to cavitation erosion from aluminum alloy 6082.

Sample	State	Diameters of Surfaces Subject to Cavitational Erosion					Maximum Penetration Depth of Cavitational Erosion	
		Outer Diameter	Intermediate Diameter		Inner Diameter		Stereo Measured	Optically Measured
		μm	μm	%	μm	%	μm	μm
**U**	T651	14,359	12,213	85	10,593	74	490.87	490
**UOP**	T651 + 140 °C/1 h	14,439	11,496	80	8609	60	251	253.35
**UOL**	T651 + 140 °C/12 h	14,286	11,712	82	8529	60	249	248.83
**UOI**	T651 + 140 °C/24 h	14,433	12,022	83	7765	54	215	212.63
**UP**	T651 + 180 °C/1 h	15,661	12,847	82	11,161	71	341	337.25
**UL**	T651 + 180 °C/12 h	14,373	11,672	81	9988	69	335	314.63
**UI**	T651 + 190 °C/24 h	14,398	12,130	84	9574	66	267	266.92

## Data Availability

Not applicable.
